# Informational Theory of Aging: The Life Extension Method Based on the Bone Marrow Transplantation

**DOI:** 10.1155/2015/686249

**Published:** 2015-09-30

**Authors:** Alexey V. Karnaukhov, Elena V. Karnaukhova, Larisa A. Sergievich, Natalia A. Karnaukhova, Elena V. Bogdanenko, Irina A. Manokhina, Valery N. Karnaukhov

**Affiliations:** ^1^Institute of Cell Biophysics, Russian Academy of Sciences, Institutskaya Street 3, Pushchino, Moscow 142290, Russia; ^2^Institute of General Pathology and Pathophysiology, Russian Academy of Medical Sciences, Baltiyskaya Street 8, Moscow 125315, Russia

## Abstract

The method of lifespan extension that is a practical application of the informational theory of aging is proposed. In this theory, the degradation (error accumulation) of the genetic information in cells is considered a main cause of aging. According to it, our method is based on the transplantation of genetically identical (or similar) stem cells with the lower number of genomic errors to the old recipients. For humans and large mammals, this method can be realized by cryopreservation of their own stem cells, taken in a young age, for the later autologous transplantation in old age. To test this method experimentally, we chose laboratory animals of relatively short lifespan (mouse). Because it is difficult to isolate the required amount of the stem cells (e.g., bone marrow) without significant damage for animals, we used the bone marrow transplantation from sacrificed inbred young donors. It is shown that the lifespan extension of recipients depends on level of their genetic similarity (syngeneity) with donors. We have achieved the lifespan increase of the experimental mice by 34% when the transplantation of the bone marrow with high level of genetic similarity was used.

## 1. Introduction

In the present work, the possibility of increasing the lifespan of mammalians and humans by transplantation of the genetically identical (or similar) stem cells with the lower number of the genomic errors to the old recipients is investigated. The idea of this method follows directly from informational theory of aging [1] in which the degradation (error accumulation) of the genetic information in cells is considered a basic reason of aging (see more detailed information in Discussion). Informational theory of aging links the accumulation of genomic errors in cells with a decrease of their functionality, which in turn leads to decreasing functionality of whole organism (vitality) and finally to increasing probability of its death.

It is well known that the stem cells are a source for many different types of cells. As a result of the transplantation of the stem cells of young donors with the low number of genomic errors the increase of the number of various cells with the reduced number of genomic errors and improving of the overall functionality of the organism (vitality) can be expected.

However, where can one get the genetically identical stem cells with the lower number of the genomic errors? The cryopreservation of the animal's (human's) own stem cells that have been isolated at a young age is one of the possible techniques. Indeed, it seems obvious that in the young cells stored in cryogenic conditions the number of genomic errors will be lower than in the cells of the old organism. As the source of the various types of stem cells (mesenchymal and hematopoietic) the bone marrow (BM) can be used.

**Figure 1:**
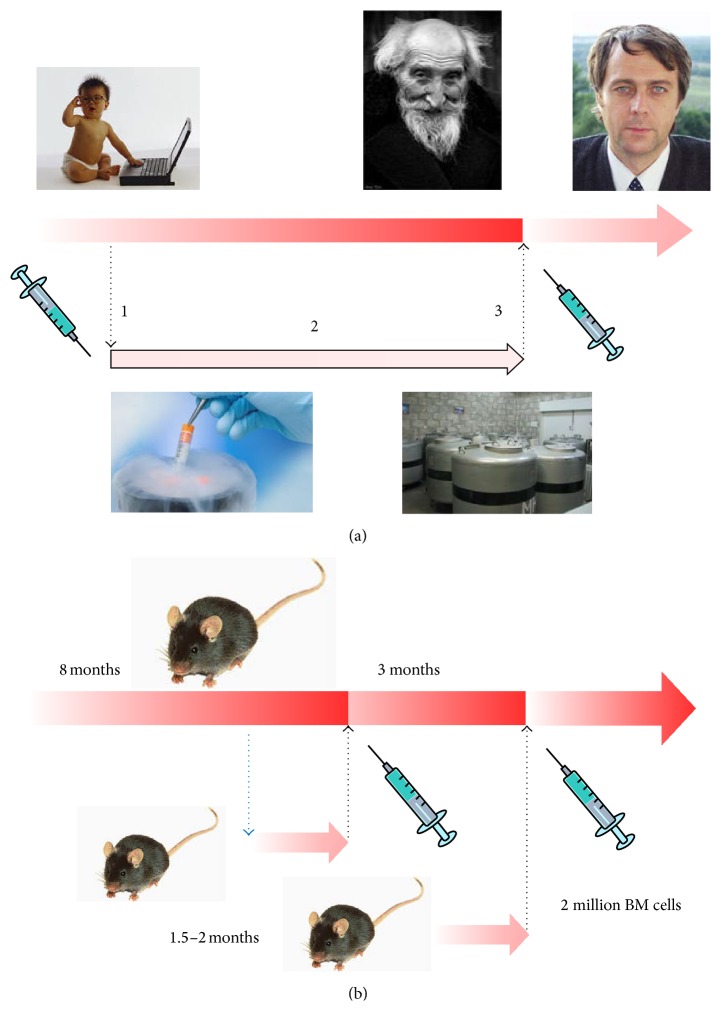
Scheme illustrates the method of rejuvenation for human: (a) 1: isolation of bone marrow (BM), 2: BM cryopreservation, 3: BM autotransplantation in old age; (b) experimental scheme of BM syngeneic transplantation to old mice from young donor mice every 3 months.

It is necessary to emphasize that all the basic operations of this variant of our method (Figure 1):

1. isolation (collection) of BM,
2. cryopreservation of BM,
3. BM autotransplantation to recipient in old age,

are well established procedures, with many years of application experience and are approved for medical use in most of countries [2–7].

Unfortunately, a very long time would be required for the direct verification of this method for large animals and humans because of their large initial life expectancy. Use of laboratory animals with the small lifespan (mice and rats) is faced with a problem of another kind. Because of the small size of these animals it is difficult to isolate (take) their BM without significant damage to their health.

This reason has forced us to modify the scheme of our experiment. Instead of application of the autologous transplantation (Figure 1(a)) we used the BM transplantation from genetically similar animals of inbred groups (Figure 1(b)) with the various levels of genetic similarity (syngeneity).

Such scheme of experiments gives the following additional possibilities: first, we can study the increase of lifespan as the function from the level of genetic similarity (syngeneity) of donors and recipients and second the engraftment of the transplanted cells can be observed if the transgenic animals are used (GFP^+^ mice).

## 2. Materials and Methods

### 2.1. Animals

The mouse strain C57BL/6-Tg(ACTBEGFP)1Osb/J carries a transgene of an enhanced green fluorescent protein in chromosome 15 (briefly eGFP C57BL/6 strain) and was received with the assistance of A. M. Malashenko (Scientific Center of Biomedical Technologies, Russian Academy of Medical Sciences) from Jackson Laboratory, Bar Harbor, United States, and thanks to the kind permission of A. V. Chervonskii it was used in the experiment.

In the eGFP C57BL/6 mouse strain, some mice carry a green fluorescent protein gene (eGFP^+^ C57BL/6 mice), which is a vital label allowing unambiguous identification of donor cells following their transplantation into the recipient organism that does not carry this gene [8, 9]. Mice carrying the green fluorescent protein gene were selected as donors for performing both syngeneic and allogeneic transplantation. When syngeneic transplantation was performed, the donor-recipient pair was taken from the same strain, with the recipients being mice not carrying the eGFP transgene (eGFP^−^ C57BL/6 mice); moreover, the donor and recipient were of the same sex. When allogeneic transplantation was performed, female mice of the BALB strain were used as recipients.

Mice were kept in the vivarium of the Institute of Cell Biophysics, Russian Academy of Sciences, in standard conditions. Granulated food for rodents supplemented with vitamins was used for feeding. The animals were additionally fed with grain mix consisting of wheat, barley, red millet, sunflower seeds, corn, and grass granules.

### 2.2. Isolation and Transplantation of BM

The donor mice were euthanized using the cervical dislocation technique. BM was isolated from two femur bones via grounding in a porcelain mortar with a phosphate saline buffer (600 *μ*L). The obtained suspension was filtered through a nylon filter with a pore size of 70 *μ*m. Transplantation was performed by administering of 100 *μ*L (2 × 10^6^ cells) of the whole fraction of BM immediately after its isolation into a side tail vein with an insulin syringe. The tail of the animal was preheated at 40–45°C with warm water.

### 2.3. Gerontology Experiment on Effect of Syngeneic BM Transplantation on the Lifespan of Mice

To study the problem, how the level of genetic similarity (syngeneity) of donors and recipients influences the lifespan extension, three groups of mice within inbred strain eGFP C57BL/6 were formed on the principle of varying degrees of syngeneity. The fourth group of mice with minimal level of genetic similarity was formed for allotransplantation (BALB mice were recipients and eGFP^+^ C57BL/6 mice were donors).

For syngeneic transplantation, we formed three experimental groups of eGFP^−^ C57BL/6 mice. Females were as the recipients and donors in two groups and in the third group males were as recipients and males and females were as donors. In three control groups of eGFP^−^ C57BL/6 mice BM transplantation was not performed. The number of animals was 11, 10, and 21 mice in the first, second, and third experimental groups and 12, 13, and 12 mice in three control groups, respectively.

Before transplantation works, additional inbreeding within the first experimental and control groups was carried out. We obtained progeny of 3-4 subsequent generations from the same female and her young male offspring or descendant of next 2-3 generations to reach high level of syngeneity. In the second and third experimental and control groups additional inbreeding was not carried out.

For the first and second experimental groups, donors were selected within offspring of the recipient mouse. The females of the experimental group were mated with male mouse (brother or son) to obtain offspring with a high degree of inbreeding, starting at 5–8 months of age with intervals of 3 months. From the resulting litter, eGFP^+^ 1.5–2-month-old donors for these mice were chosen. For old females who were unable to produce offspring, the young eGFP^+^ donors, the descendants of these females of the second and subsequent generations, were selected.

In the control groups of females, half of the animals produced 3-4 litters during lifetime.

For the third experimental group consisting of males, we have chosen as donors the males and females of 1.5–3 months carrying the gene of green fluorescent protein regardless of the relationship degree.

Thus, the first experimental group had the highest level of syngeneity between the donor of BM and the recipient (because of additional inbreeding procedure and choosing the donors among progenies of the females). The second group had the middle level of syngeneity (there was no additional inbreeding but the donors were progenies of the females). The third group consisting of males had the minimal level of syngeneity (the donors were not progenies of the males).

In all three experiments, BM transplantation (2 × 10^6^ cells) was performed with the intervals of 3 months to the death of the animal. Experiments were started at age of 7-8 months for the recipients of the first and the second experimental groups and at age of 6–10 months for those of the third group.

### 2.4. Gerontology Investigation of Effect of Allogeneic BM Transplantation on the Lifespan of Mice

Experiment was carried out using 1.5–3-month-old eGFP^+^ C57BL/6 mice as donors and 13-14-month-old BALB mice (14 females) as recipients. Transplantation of BM in the amount of 2 × 10^6^ cells was performed periodically, starting from 13-14 months of age, and repeated every 3 months until the death of the animal. The BALB/c mice (14 females) were used in the control group, for which transplantation was not performed.

### 2.5. Experiments on the Engraftment of BM Cells

To assess the engraftment of eGFP^+^ BM cells, the dynamics of eGFP^+^ cell numbers in recipient BM, spleen, thymus, and blood by fluorescence were examined.

In syngeneic transplantation experiments within inbred eGFP C57BL/6 strain, the 1.5–2-month-old eGFP^+^ mice were used as donors for eGFP^−^ young group (3-4-month-old recipients) and 1.5–3-month-old eGFP^+^ donor mice were used for eGFP^−^ old group (10–14-month-old recipients). In experiments on allogeneic transplantation, 1.5–2-month-old eGFP^+^ C57BL/6 mice were used as donors for 3-4-month-old BALB mice.

The investigation was carried out for 14–17 days following BM transplantation in the syngeneic transplantation experiments and for 7 days in the experiments on the allogeneic transplantation until eGFP^+^ cells were detectable in the investigated organs. Each point represented an average value obtained from two to six animals.

The recipient mice were euthanized using the cervical dislocation technique after certain times following the BM administration. During autopsy, thymus and spleen were placed to phosphate saline buffer, washed, and weighed. Next, organs were pulped with a scalpel through a 70 *μ*m pore size nylon filter into a 1 or 2 mL volume of the phosphate saline buffer for the thymus and spleen, respectively. A 60 *μ*L aliquot of blood was collected from thoracic cavity into a tube containing 15 *μ*L of 0.5 M EDTA. BM was collected from one of the femurs using the technique described above. 15 *μ*L aliquot of blood, suspension of BM, thymus, and spleen were placed onto microscope slides coated with polylysine (Thermo Scientific, Germany) and covered with a 20 × 20 mm cover slips. The presence of eGFP^+^ cells in them was revealed with an AxioCam Z1 fluorescence microscope equipped with an AxioCam MRc5 color digital camera (Carl Zeiss, Germany). The eGFP protein has a fluorescence maximum at 508 nm in the green region of the spectrum when illuminated with light in the wavelength region of 395–475 nm.

For estimation of the eGFP^+^ cells quantity in the sample, they were counted on eight horizontal lines from the left to the right edge of the cover slip uniformly distributed within the sample. The number of the cells was estimated using arbitrary units from 1 to 5 (1: from 5 to 40 cells; 2: from 41 to 160 cells; 3: from 161 to 400 cells; 4: from 401 to 600 cells; and 5: more than 600 cells in the sample).

### 2.6. Immunofluorescence

For staining of cell surface proteins, BM or spleen cell suspensions isolated using the technique described above were incubated with phycoerythrin- (PE-) coupled antibodies against mouse CD117 (BioLegend, San Diego, CA, USA) and CD45R/B220 (BD Biosciences, San Jose, CA, USA) for 1 h at 4°C in the dark. Then cells were washed in PBS and fluorescence was observed microscopically.

### 2.7. Statistical Data Processing

The SigmaPlot 12.0 software package was used. Confidence interval for the difference in the mean lifespan of mice between the control and test groups was determined using a standard Student's *t*-test [10]. For approximation of experimental data, polynomial functions of the first–third order calculated with the help of the MATEMATIKA 5.2 computer language were used.

## 3. Results

### 3.1. Effect of Syngeneic BM Transplantation on the Lifespan of Mice

In the present study three groups of experimental animals for the syngeneic transplantation (two consisted of females and one consisted of males) were formed. Experiments were designed to reduce the immune response to a donor's cell as result of some variation of alleles of non-major histocompatibility complex genes. For this purpose, BM cells were isolated from the young offspring of the first and subsequent generations of the recipients. Transplantation has been repeated every 3 months until the death of recipients.

**Figure 2:**
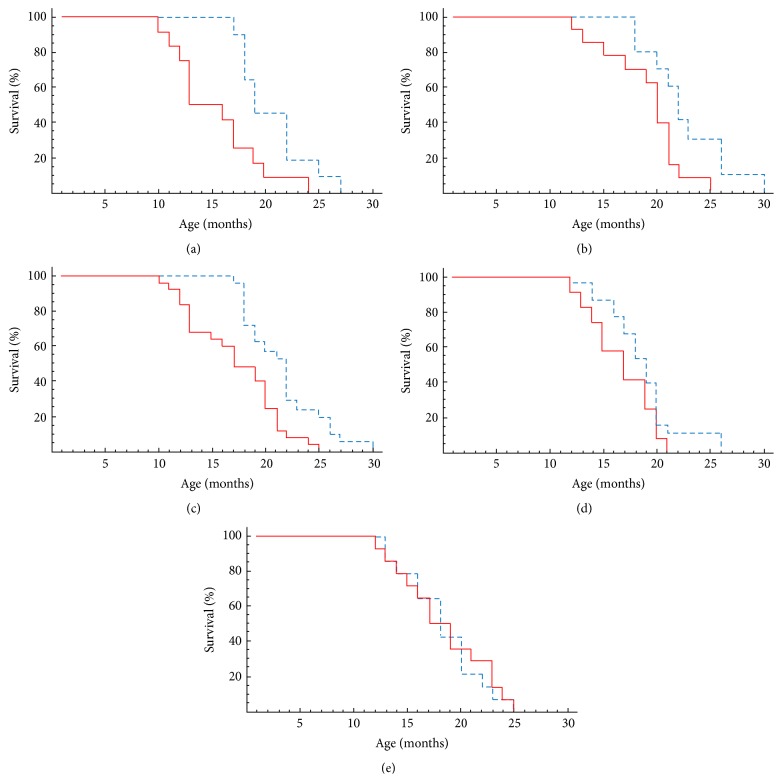
Dependence of survival of the experimental and control group of mice on age (months). Experimental group (dashed line) was transplanted without additional conditioning with 2*∗*10^6^ bone marrow cells from young donors starting with recipient age of 6–8 months (syngeneic transplantation (a)–(d)) or 13-14 months (allogeneic transplantation (e)), regularly every three months. Solid line: control group (no transplantation). (a) First group (eGFP C57Bl/6 female mice for which the additional inbreeding was carried out to approximate genomes of donor and recipient): syngeneic transplantation to eGFP^−^ mice from eGFP^+^ mice. Mean lifespan (MLS) was 20.6 ± 2.2 months (experiment, *n* = 11) and 15.4 ± 2.6 months (control, *n* = 12) (34% excess of control; *P* = 0.05). (b) Second group (eGFP C57Bl/6 female mice): syngeneic transplantation to eGFP^−^ mice from eGFP^+^ mice. MLS was 22.6 ± 1.6 months (experiment, *n* = 10) and 18.9 ± 1.4 months (control, *n* = 13) (19% excess of control; *P* = 0.20). (c) First and second groups together: MLS for the experimental group was 21.5 ± 1.6 months and for the control – 17.2 ± 1.8 months (25% excess of control; *P* = 0.05). (d) Third group: syngeneic transplantation to eGFP^−^ C57Bl/6 mice (males) from eGFP^+^ C57Bl/6 mice (females and males); MLS was 18.6 ± 0.6 months (experiment, *n* = 21) and 15.4 ± 2.6 months (control, *n* = 12) (10% excess of control; *P* = 0.40). (e) Allogeneic transplantation to BALB mice (females) from eGFP^+^ C57Bl/6 mice (females and males): MLS was 18.3 ± 2.2 months (experiment, *n* = 14) and 18.4 ± 2.4 months (control, *n* = 14) (*P* = 0.05; significant difference was not observed in survival dynamics of experimental and control groups of BALB mice).

On Figures 2(a) and 2(b) the dependence of survival on age of the first and the second experimental and control groups of mice is presented. For the first experimental group of mice, the mean lifespan is 34% longer (20.6 ± 2.2 versus 15.4 ± 2.6 months; *P* = 0.05) and for the second experimental group the mean lifespan is 19% longer than for the control ones (22.6 ± 1.6 versus 18.9 ± 1.4 months; *P* = 0.20). It should be noted that such difference in the increment of mean lifespan between the first and the second experimental groups is a natural result and can be explained by previously carried out additional inbreeding within the first group of mice.

On Figure 2(c) the survival curves for the first and second experimental groups together and total control groups are shown. The mean lifespan in the experiment was 21.5 ± 1.6 months, that is, 25% more than in the control group (17.2 ± 1.8 months; *P* = 0.05). The combined data allow us to improve the statistical reliability of the result, obtained by increasing the number of animals in the groups, and to draw a conclusion about the possibility of a significant extension of the lifespan of animals with syngeneic BM transplantation.

For the third experimental group, increase of the lifespan value reached only 10% and was 18.6 ± 0.6 months compared with 16.8 ± 0.8 months in the control group (*P* = 0.4) (Figure 2(d)).

### 3.2. Effect of Allogeneic BM Transplantation on the Lifespan of Mice

The dependence of the BALB mice survival on age after allogeneic BM transplantation (eGFP^+^ C57Bl/6 mice are as donors) is presented in Figure 2(e). It is evident that the dynamics of survival of the experimental and control groups differ slightly. There is no significant difference between their mean lifespans. They reach 18.3 ± 2.2 months for the experimental group and 18.4 ± 2.4 months for the control group (*P* = 0.05).

### 3.3. Determination of Engraftment Degree of Donor Cells after Transplantation

**Figure 3:**
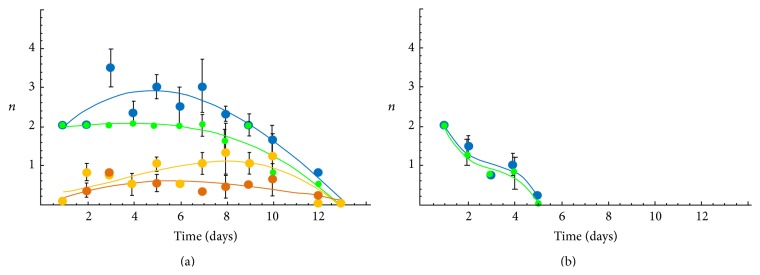
Dependence of the number of eGFP^+^ cells in organs of blood system (BM, spleen, thymus, and blood) of the recipient mice on time after BM transplantation. (a) eGFP C57Bl/6 strain (syngeneic transplantation); (b) BALB strain (allogeneic transplantation) (green: BM, blue: spleen, yellow: thymus, and orange: blood). The abscissa axis indicates time after transplantation in days; the ordinate axis indicates number of eGFP^+^ cells (*n*: arbitrary units). Solid lines represent a cubic polynomial approximation. (Error: mean ± SD, the mean of values of 2–6 experiments per point).

To determine the engraftment degree of donor cells in the host organism, quantity of eGFP^+^ cells was estimated in BM, spleen, thymus, and blood of 3-4-month-old recipient mice on different days after transplantation of BM suspension (2 × 10^6^ cells). The dependence of change of the eGFP^+^ cells amount in mice organs on time after syngeneic and allogeneic transplantation is presented in Figure 3.

The maximum time of eGFP^+^ cells engraftment was established after syngeneic transplantation and on the 12th day the donor cells are still registered in BM, spleen, and thymus (Figure 3(a)). After allogeneic transplantation, the maximum stay of eGFP^+^ cells was 5 days for the BALB strain (Figure 3(b)). After the syngeneic and allogeneic transplantation, the greatest number of donor cells was recorded in BM and spleen. In thymus and blood, after allogeneic transplantation, for the first 2-3 days only individual cells were recorded. In older recipients (10–14-month-old) after the syngeneic transplantation, the engraftment dynamic of the blood system was similar to that for young recipients, and the maximum time of registration of donor cells (17 days) exceeded that of the young group by 40% (not shown in the figure).

**Figure 4:**
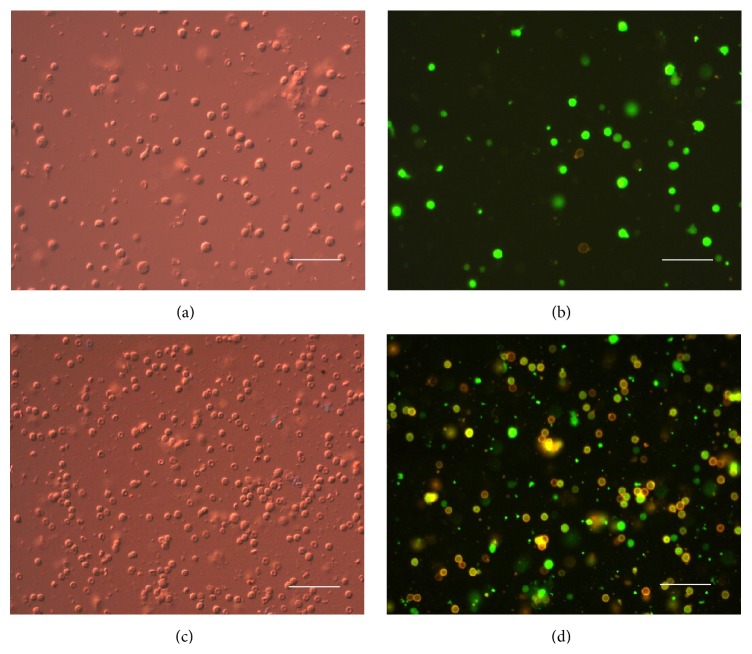
Recipient cells after syngeneic transplantation of eGFP^+^ cells at the time of maximal colonization (magnification ×40; scale bars represent 50 *μ*m). (a, b): BM cells: (a) differential interference contrast (DIC) microscopy and (b) fluorescence image: green: eGFP^+^ cells, orange: immunofluorescence staining with phycoerythrin- (PE-) coupled antibodies showing CD117 (hematopoietic progenitor's marker) expression on host cells. (c, d) Spleen cells: (c) DIC and (d) fluorescence image: green: eGFP^+^ cells, orange: immunofluorescence staining with phycoerythrin- (PE-) coupled antibodies showing CD45R/B220 (B lymphocyte marker at all stages) expression on donor and host cells.

In Figure 4, the cells of BM (a, b) and spleen (c, d) in the recipient at the time of maximum engraftment are presented. The differential interference contrast (DIC) microscopy (a, c) shows all the cells in the suspension. Only host hematopoietic progenitor cells (CD117 expression, orange fluorescence) are visible in BM (Figure 4(b)). In the spleen both donor and host cells highly express CD45, which are revealed with bright orange fluorescence (Figure 4(d)).

## 4. Discussion

### 4.1. Discussion of Results

The main experimental result of this work is finding the relation between the lifespan increase and genetic similarity (syngeneity) of donors and recipients within the framework of the proposed method. The value of lifespan increase in our experiments varies from 34% for high-level syngeneic transplantation to 0% for allogeneic transplantation. One can note that our results are in good agreement with the results of earlier works [11–18] (Table 1), where the transplantation with the low levels of syngeneity is used.

It seems important to explain these experimental results within the framework of our informational theory of aging in more detail. Hence, the main points of this theory need to be described here.

There are many experimental works in which the fact of accumulation of lesions in the genetic material of the cells of various organisms in the course of aging is directly established [19–22].

However, the absence of a clear answer to the question why the genomic errors do not accumulate in germ line cells from generation to generation [23–28] induces search for other basic mechanisms of aging, such as epigenetic, that are not related with the genome damage by random factors [29–35].

**Figure 5:**
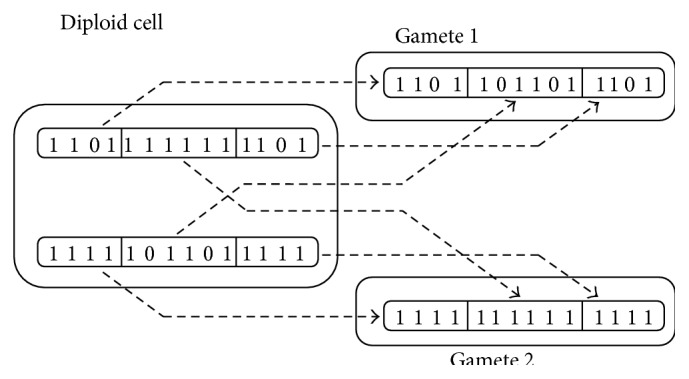
Example that demonstrates the possibility of formation of gametes with a lover density of genetic errors, compared with the original diploid germ line cells (damaged gene: “0” and normal gene: “1”). In gamete 2, all genes are normal.

In 2009 we proposed [1] a solution for the problem of “escaping germ line from aging” within the framework of a wide range of hypotheses based on the aging notion as a process of accumulation of genomic errors [24–27, 36–38]. This solution turned out to be quite simple. It has been found that the mechanism providing “correction” of genomic errors in germ line cells involves two elements that are well known and common practically for all eukaryotic organisms.

(*1) Gene Recombination (Crossing Over)*. It is well known that as a result of gene recombination (crossing over) in cells of germ line the haploid daughter cells-precursors are replicated. These haploid daughter cells (gametes) contain 1/2 of the genetic information of next generation organism. We need to note here that the density of gamete's genomic errors differs from the density of genomic errors in germ line cells of maternal organism. Indeed, the stochastic nature of the location of genomic errors and the stochastic nature of gene recombination (crossover) leads to the random distribution of errors between gamete's genomes as shown in Figure 5. In addition, it is important that the density of the genomic errors in some of them be lower than in the cells of germ line of maternal organism (e.g., gamete 2 in Figure 5).

(*2) Gamete Selection*. The second element of the given mechanism of “correction” of the genomic errors is the process of selection of precursor cells (gametes) with minimal error density. This is possible due to the excessive amount of gametes. Moreover, the haploidy of such cells raises the efficiency selection inasmuch as it elevates the specific contribution of every error into the overall reduction of gamete functionality.

Although each element of this mechanism (gene recombination + gamete selection) was in itself well known and studied, the conclusion that the reduction of the density of genomic errors comes out from their joint action was a priority of our previous works. This solution of the paradox of the “not aging germ line” rehabilitates a wide range of aging hypotheses united by the idea of degradation of the genetic information by the aging [24–27, 36–38].

The conception of aging as the process of accumulation genomic errors (degradation of the genetic information) in the most general case, supplemented with the above described mechanism of “correction” genetic lesions in germ line cells, has the name of an informational theory of aging.

**Figure 6:**
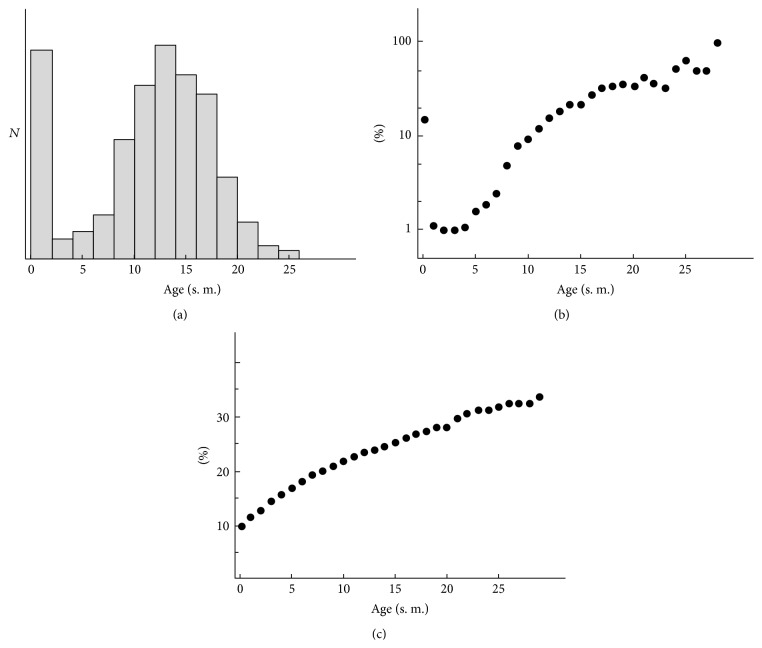
Age-related mortality and genomic damages in model population of multicellular organisms at the stage of dynamic equilibrium: (a) absolute, (b) relative to group size, and (c) mean percentage of damaged genes. Age is represented in steps of model (s. m.).

To test the workability of the informational theory of aging, we have built the simple simulation model [1]. In spite of simplicity of this model, it correctly describes the basic properties of such a complicated process as aging. Those phenomena are reproduced in the model:

- the mortality curve for model organisms (Figure 6(a));
- Gompertz curve [38] (Figure 6(b));
- accumulation of the genomic errors in old age (Figure 6(c)).

It should be noted here that the Gompertz curve describes aging dynamics of wide class of eukaryotic organisms from the colonies of yeast up to the higher mammals including humans. This fact demonstrates simplicity of the aging mechanisms in their basis.

It is interesting that the model reproduces not only the linear part of the Gompertz curve but also the phenomenon of “neonatal mortality” and the phenomenon of relative stabilization of mortality in elder age groups.

### 4.2. Formulation of the Model and Comments

The basis of the model is a population genome matrix $\tilde{G}(t)=G(i,p,m,s,t)$; the element is taking the value of 1 if the gene is intact or 0 if it contains an error; the indices numerate the organism in the population *i* = (1,…, *ni*), the cell in the organism *p* = (1,…, *np*), the gene in the genome *m* = (1,…, *nm*), and the genome copy in the diploid cell *s* = (1, 2); *t* is the discrete time parameter.

This matrix evolves in time in accordance with three main algorithms:

- aging (accumulation of errors in genomes);
- death of old age (upon exceeding a certain threshold of genomic errors);
- birth of a new organism (rejuvenation of genome).

(1)* Aging* (defect accumulation) is realized as follows: *M* quartets {*i* ^*∗*^, *p* ^*∗*^, *m* ^*∗*^, *s* ^*∗*^}  are randomly chosen at every modeling step *t*, and the corresponding genes are considered to be defective:

(1) $$\begin{array}{c} G\left(\;i^{*},p^{*},m^{*},s^{*},t+\Delta t\;\right)=0. \end{array}$$

The other elements of $\tilde{G}(t)$ retain their values. Parameter *M* specifies the rate of error accumulation.

An important feature of the model (it explains the use of the term “informational theory of aging”) is the absence of mechanisms able to correct the informational errors in the genome. It is different, in principle, from the more general class of genome damage for which the molecular mechanism of correction (repair) may exist.

(2)* Death* of model organism happens when the number of its functional cells falls below a critical level *N* ^*a*^ < *N*_min_^*a*^. In the model, loss of cell function occurs when even one gene has a defect in both copies of genome. That is, cell *p*_0_ of organism *i*_0_ is considered nonfunctional if, for at least one gene *m*,

(2) $$\begin{array}{c} G\left(\;i_{0},p_{0},m,1,t\;\right)=G\left(\;i_{0},p_{0},m,2,t\;\right)=0. \end{array}$$

Upon death of *i*_0_, the corresponding block $\tilde{G}(t)$ is replaced with a block for a newly born organism. In this way, the total number of simulated organisms in the population is held constant.

(3)* Birth* of a new organism involves several steps as follows:

(3.1) A pair of parental cells (*i*_1_, *p*_1_), (*i*_2_, *p*_2_) is randomly chosen among viable cells of different organisms (*i*_1_, *i*_2_) above a certain age *T*_min_.
(3.2) Each of parental cells produces an even number 2*∗nj* of gametes with a single set of genes (formulae for both cells are identical):
  (3) G~gi1,p1=Ggi1,p1m,j=Gi1,p1,m,sj,m,tGgi1,p1m,j+nj=Gi1,p1,m,3−sj,m,t,
 where *j* = (1,…, *nj*). The random matrix *s*(*j*, *m*), taking values (1, 2), determines crossover procedure in the model. It maps diploid genome of the parents on haploid genome of gametes. Figure 5 shows the 14-gene diploid genome of parent with four errors (two in each set). Gene recombination (crossover) (*s*(1, *m*) = {1,1, 1,1, 2,2, 2,2, 2,2, 1,1, 1,1}, *nj* = 1) yields two gametes: one with all four errors (*j* = 1) and the other without any (*j* = 2).
(3.3) Upon forming two sets of gametes with genomic matrices ${\tilde{G}}_{g}^{i_{1},p_{1}}$ and ${\tilde{G}}_{g}^{i_{2},p_{2}}$, one gamete from each set is chosen with a probability proportional to the viability of this gamete's *V* ^*i*_1_,*p*_1_^(*j*) and *V* ^*i*_2_,*p*_2_^(*j*), which itself depends on the number of genomic errors as (identically for *V* ^*i*_2_,*p*_2_^(*j*))
  (4) $$\begin{array}{c} V^{i_{1},p_{1}}\left(\;j\;\right)=\exp\left\{\;\alpha \overset{nm}{\underset{m=1}{\sum }}\left(\;G_{g}^{i_{1},p_{1}}\left(\;m,j\;\right)-1\;\right)\;\right\}, \end{array}$$
 where  *α*  is a parameter specifying the extent to which genomic damage affects gamete viability,  *j* = (1,…, 2*nj*). In essence, this defines a process of selection of gametes for the minimal amount of errors: ${\tilde{G}}_{g}^{i_{1},p_{1}}\to j_{1}$; ${\tilde{G}}_{g}^{i_{2},p_{2}}\to j_{2}$, where  *j*_1_, *j*_2_  denote the gametes obtained from parents *i*_1_, *i*_2_.
(3.4) Finally, the matrices of the two selected «mating» gametes  (*j*_1_, *j*_2_)  are combined in a new block of the population matrix to represent the organism that replaces the dead  *i*_0_:
  (5) $$\begin{array}{c} G\left(\;i_{0},p,m,s,t+\Delta t\;\right)=G_{g}^{i_{s},p_{s}}\left(\;m,j_{s}\;\right). \end{array}$$

When all organisms that died within the given time step (*t* → *t* + Δ*t*) have been replaced with newborn ones (which technically means the formation of a new population genome matrix and zeroing of the corresponding age counters), the model goes to the next time step (*t* + Δ*t* → *t* + 2Δ*t*).

**Figure 7:**
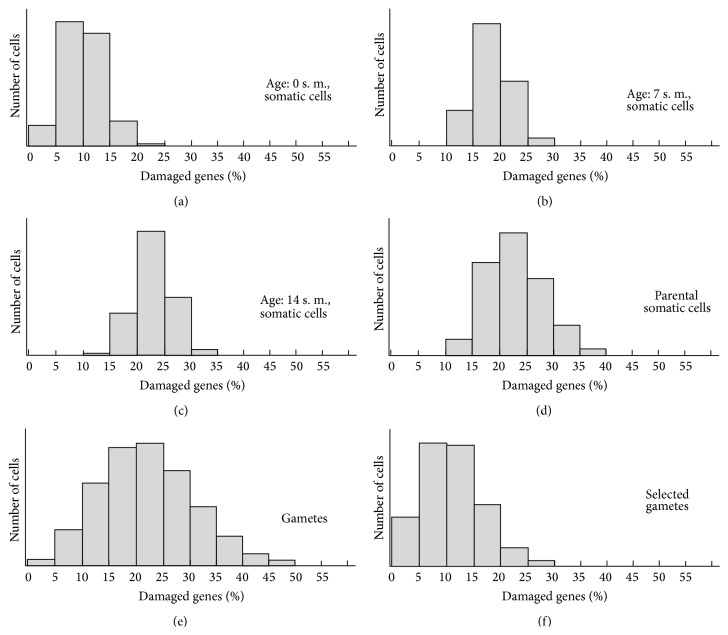
Distribution of the percentage of damaged genes for different age groups and cell kinds. Age is represented in steps of model (s. m.).

Once the model is formulated, the process of accumulation and correcting of genomic errors can be viewed in more detail.  Figure 7 displays the steady-state distribution of the number of genomic errors (nonfunctional genes in percentage) for cells of different age groups and type.

As noted above, this simple model correctly reproduces the survival curve (Gompertz curve) for the eukaryotic organisms (Figure 6). It should be noted that within the framework of the model that has been formulated we could not only reproduce known results but also perform such experiments in the model populations that are difficult (impossible) to carry out in real system. For example, we can “turn off” the process of gene recombination (crossing over).

**Figure 8:**
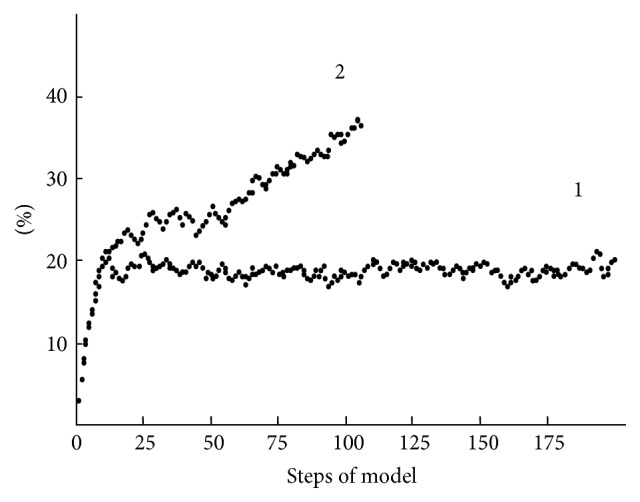
Accumulation of genomic damages over time in model populations with operative (1) and blocked (2) crossing over.

The key role of gene recombination during gametogenesis for stabilizing of the model's population is demonstrated in Figure 8.

The steady-state with a 20% level of the genomic errors is reached in 10–15 of time steps in the model with the active algorithm of the gene recombination (crossover) and can be maintained indefinitely long (curve 1). In model in which the algorithm of the gene recombination is blocked, the model's population will end quite soon (110 of time steps) because of accumulation of genomic damage (curve 2).

Unfortunately, this model is too simple to explain the results of our experiments, in which the rise on 3-4 months of life expectancy of laboratory animals has been observed (Table 1), whereas bone marrow cells were detected in the recipient only about 0.5 months (Figure 3). On the other hand, the total residence time of donor cells, considering the number of bone marrow injection, was about 2.5 months (5 injections *∗* 0. 5 months), which is much closer to the experimental values of lifespan increase on 3-4 months.

To understand this fact, we need to develop the more advanced model within the framework of the informational theory of aging.

### 4.3. The Project of the More Advanced Model

The more general description will include the following principals (not the full list):

(1) In somatic cells of eukaryotic organism, each of the two copies of genetic information ${\hat{G}}_{1}^{p}\left(t\right),{\hat{G}}_{2}^{p}\left(t\right)$ throughout the cell lifespan accumulates defects (errors):
  (6) RG^1pt+Δt≥RG^1pt;RG^2pt+Δt≥RG^2pt,
 where $R({\hat{G}}_{s}^{p}(t))$ is the number of defects in the genomic copy *s* = (1, 2) of somatic cell  *p*  at a moment  *t*.
(2) After a mitotic division *p*′ → *p*, *q* the number of defects in daughter cells *p*, *q* will be not smaller than in mother's *p*′:
  (7) RG^spt+Δt≥RG^sp′t;RG^sqt+Δt≥RG^sp′t.
(3) Accumulation of the genomic errors $R^{\Sigma }\left(\left\{{\hat{G}}_{s}^{p^{\prime }}(t)\right\}\right)$ decreases the viability of individual cells $V^{p}({\hat{G}}_{s}^{p}(t))$ and viability of the organism
  (8) $$\begin{array}{c} V^{\Sigma }\left(\;t\;\right)=V^{\Sigma }\left(\;\left\{\;{\hat{G}}_{s}^{p}\left(\;t\;\right)\;\right\}\;\right). \end{array}$$
(4) With viability of the organism becoming less able to withstand any adverse influence and the probability of its death *P*(*V* ^Σ^(*t*)) rising,
  (9) ddtVΣt0,∂∂VΣPVΣt0⟹ddtPVΣt≥0.

The principles that were introduced above will be the basic for wide class of models in the information theory of aging. However, the exact determination of the specific values entered in the various models may be different. The model in [1] (brief description and results are given in Section 4.2) solves the problem of demonstrating the correctness of information theory of aging in general and is greatly simplified for this purpose. It is necessary to note two important simplifications:

1. the model in [1] assumes that all cells of the organism are equivalent;
2. the functional of vitality that was inversely proportional to the amount of genomic errors is also an oversimplification.

In a more general class of models within the framework of the informational theory of aging a multicellular organism should be considered as a set of cell populations, each of which is characterized by the average number of errors *R* ^*c*^(*t*)  as well as the number of cells in the population *N* ^*c*^(*t*), where *c* indicate cell population (cluster). Alternatively, in the matrix form,

(10) R^t=Rct;N^t=Nct.

In addition, it is obvious that the vitality of the organism will depend not only on the average number of errors and on the number of cells in the corresponding cell populations but also on environmental conditions:

(11) $$\begin{array}{c} V^{\Sigma }\left(\;t\;\right)=V^{\Sigma }\left(\;\hat{N}\left(\;t\;\right),\hat{R}\left(\;t\;\right),\hat{E}\left(\;t\;\right)\;\right), \end{array}$$

where$\;\hat{E}\left(t\right)$ is matrix of environmental parameters.

Changing the number of cells in a given population, in turn, will depend on same parameters:

(12) $$\begin{array}{c} \frac{d\hat{N}\left(t\right)}{dt}=\hat{D}\left(\;\hat{N}\left(\;t\;\right),\hat{R}\left(\;t\;\right),\hat{E}\left(\;t\;\right)\;\right), \end{array}$$

where $\hat{D}\left(\hat{N}\left(t\right),\hat{R}\left(t\right),\hat{E}\left(t\right)\right)$ is matrix of dynamics of cells populations (types of cells).

Under this approach, it becomes possible to explain the results of our experiments. Indeed, donor's cell, taking some of the functions of recipient's ones, apparently allows increasing the corresponding population of recipient's cells. In this case, the increasing vitality of the organism will have prolonged nature, remaining elevated even after all the donor cells will be removed from the recipient's organism. However, in order to examine in more detail this question, it is necessary to perform additional experiments including quantitative measurements of parameters of cell populations (of hematopoietic and mesenchymal stem cells, for example) in the recipient that would define the parameters of advanced models (articles on identification of systems) [39–42].

The authors are ready to cooperate and can provide a useful guide to set up a problem and to analyze the data obtained.

### 4.4. Some Final Remarks

*Biological Aspects*. An approach presented here for explanation of aging phenomenon gives the opportunity of a fresh look at a number of well-known facts in biology and clarifies their biological sense.

1. So, for example, the well-known mechanism of gene recombination (crossing over) within the framework of our approach is not only a mechanism of optimizing the evolutionary process of eukaryotic organisms but also an important mechanism of supporting stability of their genome and preventing population degeneracy.
2. Moreover, the advantage of* eukaryotic* organization of the highly developed living forms becomes more clear. The ordinary evolutionary mechanism of individual organism elimination with genetic errors acts only when the mathematical expectation of the appearance of a genetic error during the whole life cycle is less than one.
3. Such mechanism is realized in bacteria with small genome and short life cycle. The long-lived prokaryotic organism with the complex (large) genome would unavoidably accumulate the genetic errors from the foregoing generation to the following one which would lead at last to collapse of all population.
4. Multiple repetitions of genes in the genomes of eukaryotes enhance resistance to genetic errors. We believe that the lifespan of the eukaryotic organism is longer when a degree of such repetitions is higher. The flowering plants with a potentially very long lifespan (and a large genome) are characteristic example. We consider here not only champions like sequoia or baobab but also ability of many short-lived species to reproduce themselves by vegetative way (without crossing over) during many years.
5. The role of stem cells for highly organized organisms becomes more clear.
6. It is more preferable for such organisms to have the special cells (long-term stem cells) with a decreased level of metabolism which leads to reducing the number of appearing free radicals and thus leads to decreasing rate of accumulation of genetic errors. The rate of accumulation of genetic errors in the stem cells together with the degree of repetition of genetic information determines species lifespan of different highly organized organisms (excluding a number of cases when a death of an organism has a programmed nature as the death of the salmon).

*Medical Aspects*. Conclusion about impossibility of cardinal (significant) prolongation of human life by the pharmacological ways and by methods of epigenetic rejuvenation of the own stem cells of a patient is one of the important conclusions based on the theory presented here.

Indeed, if we are to accept that genetic errors accumulation in the organism cells is the crucial factor of aging, it will be obvious that there are no biochemical procedures or chemicals which may restore the lost genetic information. In some sense our approach is one of the theoretical substantiations of a skeptical viewpoint on feasibility of rejuvenation human organism therapy [43].

At the same time, this skepticism should not be mechanically expanded on our proposed method, since it considerably differs from the earlier proposed methods and is aimed at compensation of age-specific changes associated with decreasing cell functionality, which is a common phenomenon for the completely different organisms.

*Experimental Aspects*. It should be noted that the experiments carried out by us are preliminary ones from the medical viewpoint due to experimental subject choice (mouse) and due to the obtained meaning of reliability (*P* = 0.05) which is quite sufficient for the biological researches but which is insufficient for the medical applications.

In addition, the scheme of our present experiment (Figure 1(b)) differs from the method of rejuvenation proposed for the medical application (Figure 1(a)). Therefore, the further development of the experimental investigations is of current interest.

The next concrete experiments may be proposed and may be interesting for achieving our purposes:

- carrying out works that will be analogous to these described in this paper, but in which cryopreserved bone marrow will be used;
- using relatively big mammals (rabbit, dog) to realize fully autologous transplantation (Figure 1(a));
- carrying out autologous transplantation using cloned animals (mouse);
- transplantation of the different types of the stem cells;
- induction of immune tolerance for using the donor stem cells at allogeneic transplantation.

## 5. Conclusion

The significant increase of lifespan for inbred laboratory animals demonstrates the efficiency of the proposed method for lifespan extension of multicellular organisms (human) in general and confirms the basis of the informational theory of aging. It allows developing the various methods of cellular therapy without risk of fatal consequences of immunological incompatibility and the methods of lifespan extension by using cryopreservation of stem cells taken in young age for autotransplantation in old age.

In addition, in particular we want to note two experimental facts obtained in our work:

1. the genetically not-identical bone marrow transplantation provides the significant increase of laboratory animal lifespan (up to 34%);
2. the strong dependence of the life expectancy increase from the level of genetic similarity (syngeneity) of donors and recipients exists.

From these facts the possibility of much greater increase of lifespan (than 34%) follows. It would be provided using the autotransplantation or the transplantation of the genetically identical stem cells (bone marrow) with the low level of genomic errors.

## Figures and Tables

**Table 1:** Comparison of data on influence of bone marrow (BM) and mesenchymal stem cells transplantation on the mean lifespan of mice, obtained here and in experimental works of other authors.

Number	Date	Recipients	Donors	Number of mice	Irradiation	Source and amount of cells	Mean lifespan extension (Δ)
1	1991 [11]	Mutants gus^mps^/gus^mps^, young ♀	C57Bl/6, ♀	18 (e) 91 (c)	2–4 Gy	7 *∗* 10^6^, BM (total fraction)	Δ = 197% (*P* = 0.005)

2	1991 [11]	C57Bl/6, young ♀	C57Bl/6, ♀	14 (e) 7 (c)	2–4 Gy	6 *∗* 10^6^, BM (total fraction)	Δ = −26% (*P* = 0.2)

3	2005 [12]	C57Bl/6, old ♀ (16 months)	C57Bl/6 EGFP^+^, young ♀	38 (e) 17 (c)	No irradiation	4 *∗* 10^6^, BM (total fraction) injections every 2 months	Δ = 6% (*P* = 0.4)

4	2010 [13]	BALB, old ♀ (15 months)	BALB, fetal tissue, ♂	10 (e) 10 (c)	No irradiation	10 *∗* 10^6^, fetal tissue, mesenchymal stem cells, 3 injections	Δ = 10% (*P* = 0.1)

5	2011 [14]	BALB, old ♀ (18–24 months)	C57Bl/6 EGFP^+^, young ♂	14 (e) 10 (c)	5 Gy	10^6^, mesenchymal stem cells	Δ = 16% (*P* = 0.05)

6	2011 [14]	BALB, old ♀ (18–24 months)	C57Bl/6 EGFP^+^, old ♂ (20–24 months)	10 (e) 10 (c)	5 Gy	10^6^, mesenchymal stem cells	Δ = 3% (*P* = 0.6)

7	2012 [15]	C57Bl/6, old ♀ (21.5 months)	C57Bl/6, young ♂	8 (e) 9 (c)	No irradiation	25 *∗* 10^6^, BM (total fraction) 6 injections	Δ = 6% (*P* = 0.4)

8	2013 [16]	mutants Wrn^−/−^Tre^−/−^, young ♂ (3 months)	C57Bl/6 EGFP^+^, young ♀	13 (e) 8 (c)	10 Gy	5 *∗* 10^6^, BM (total fraction)	Δ = 29% (*P* = 0.01)

9	2014 our work [17]	C57Bl/6 EGFP, old ♀ (8 months)	C57Bl/6 EGFP^+^, young ♀	11 (e) 12 (c)	No irradiation	2 *∗* 10^6^, BM (total fraction) injection every 3 months	Δ = 34% (*P* = 0.05)

10	This work	C57Bl/6 EGFP, old ♀ (8 months)	C57Bl/6 EGFP^+^, young ♀	10 (e) 13 (c)	No irradiation	2 *∗* 10^6^, BM (total fraction) injection every 3 months	Δ = 19% (*P* = 0.20)

11	This work	C57Bl/6 EGFP, old ♀ (8 months)	C57Bl/6 EGFP^+^, young ♀	21 (e) 25 (c)	No irradiation	2 *∗* 10^6^, BM (total fraction) injection every 3 months	Δ = 25% (*P* = 0.05)

12	This work	C57Bl/6 EGFP, old ♂ (8 months)	C57Bl/6 EGFP^+^, young ♀, ♂	21 (e) 12 (c)	No irradiation	2 *∗* 10^6^, BM (total fraction) injection every 3 months	Δ = 10% (*P* = 0.40)

13	2014, our work [18]	BALB, old ♀ (13 months)	C57Bl/6 EGFP^+^, young ♀, ♂	14 (e) 14 (c)	No irradiation	2 *∗* 10^6^, BM (total fraction) injection every 3 months	Significant difference is not found

♀—female, ♂—male, e—experiment, c—control.
